# Supplementary material for: Endoglin Trafficking/Exosomal Targeting in Liver Cells Depends on N-Glycosylation
Source: Cells. 2019 Aug 28;8(9):997. doi: 10.3390/cells8090997 (PMC6769735; doi:10.3390/cells8090997)
Supplement: Supplementary file 1 [file cells-08-00997-s001.pdf]

Supplementary Figures

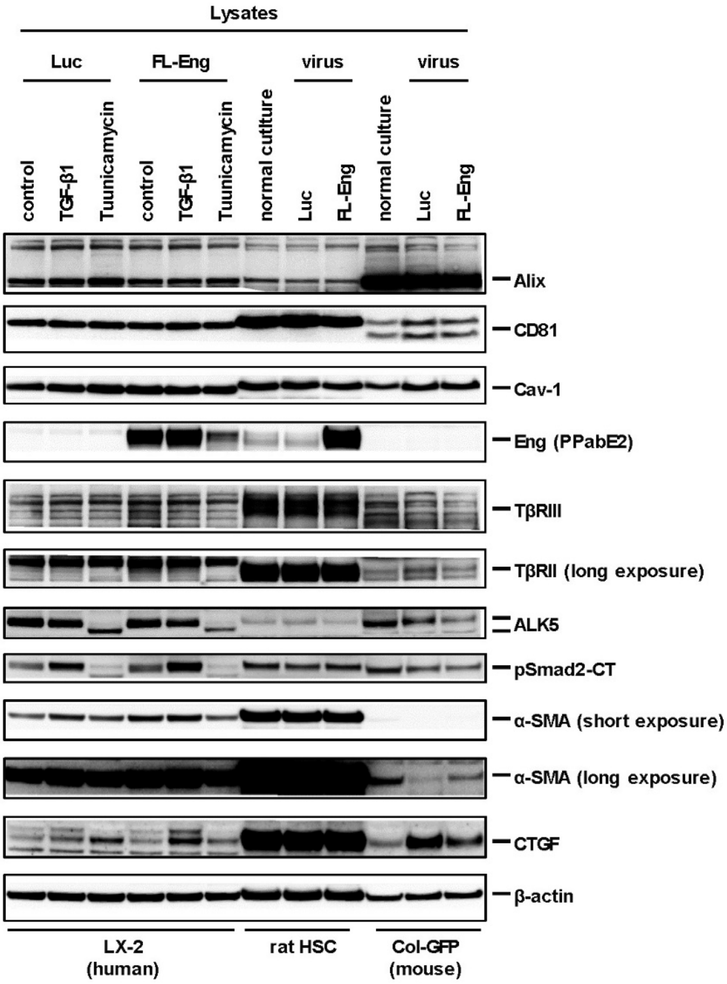

Supplementary Figure 1: Testing of antibodies in different species. Cell extracts of human hepatic stellate cells line LX-2, primary rat HSC, and murine HSC cell line Col-GFP that were infected (Luc, FL-Eng) and stimulated with TGF-β (0.1 ng/ml) or tunicamycin (0.5 μg/ml) were probed with antibodies specific for Alix, CD81, Caveolin-1 (Cav-1), Endoglin, TβRIII, TβRII, ALK5, pSmad-CT, α-SMA, CTGF, and β-actin. Control cells that were not incubated with TGF-β1 or tunicamycin were analysed in parallel to demonstrate endogenous gene expression.

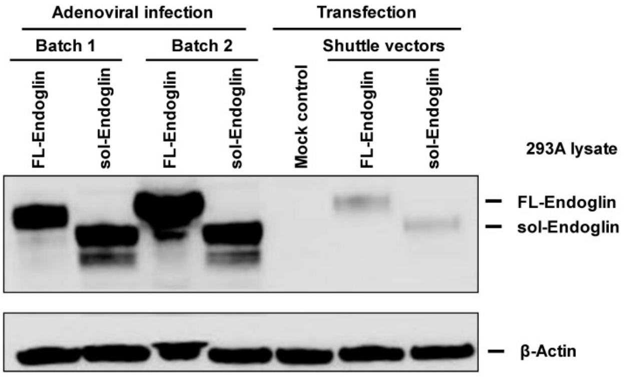

Supplementary Figure 2: Testing of adenoviral expression constructs. For verification of functionality of adenoviral constructs, 293A cells were infected with two different batches of AdEasy-1-CMV-FL-endoglin or AdEasy-1-CMV-sol-endoglin (*left panels*). As a control, cells were infected with adenoviral shuttle vectors pShuttle-CMV-FL-endoglin or pShuttle-CMV-sol-endoglin. Protein extracts were prepared and tested by Western blot for expression of FL-endoglin and sol-endoglin. Equal protein loading in each lane was verified by probing with a β-actin antibody.

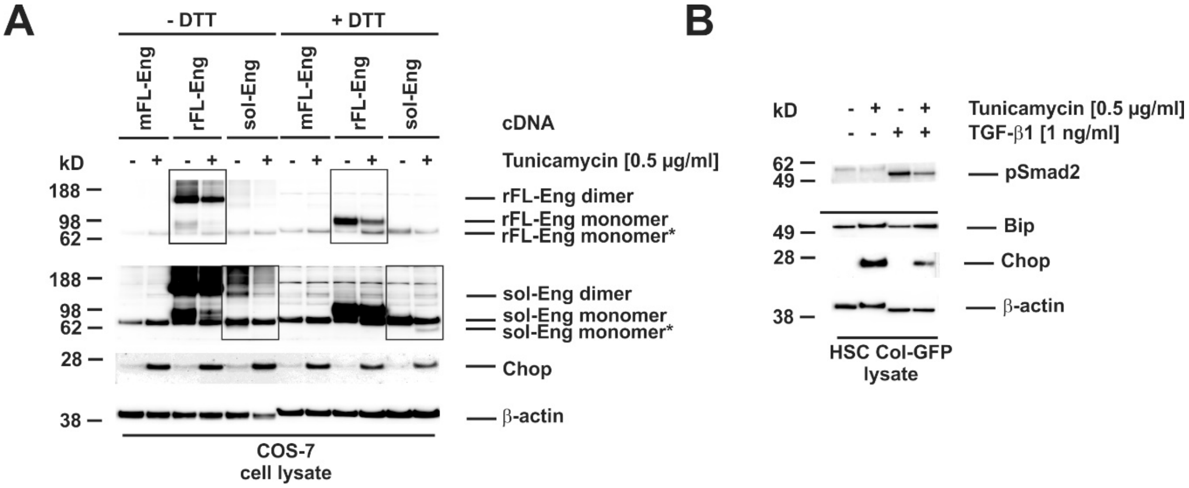

Supplementary Figure 3: Tunicamycin affects glycosylation of FL- and sol-Endoglin and induces the unfolded protein response (UPR) in COS-7 and HSC reporter cell line Col-GFP. (A) COS-7 cells were transfected with a plasmid directing expression of mouse FL-Endoglin (mFL-Eng), rat FL-Endoglin (rFL-Eng) and rat soluble Endoglin (sol-Eng). Thereafter, cells were treated with tunicamycin (0.5 µg/ml, 24 h) or left untreated. Cell extracts were prepared and examined for expression of rFL-Eng, sol-Eng and the UPR marker protein Chop in Western blot analysis. To monitor equal protein loading, the membrane was reprobed with a β-actin specific antibody. **(B)** HSC Col-GFP cells were treated or not with tunicamycin (0.5 µg/ml, 24 h) and stimulated or not with TGF-β1 (1 ng/ml; 30 min). Thereafter, proteins were extracted and analysed by Western blot for the expression and activation of the indicated proteins. Equal protein loading of the membrane was demonstrated with a β-actin specific antibody.
